# Adolescent Pregnancy and Outcomes: A Hospital-Based Comparative Study at a Tertiary Care Unit in Eastern Province, Sri Lanka

**DOI:** 10.7759/cureus.12081

**Published:** 2020-12-14

**Authors:** Markandu Thirukumar, Vijayakumary Thadchanamoorthy, Kavinda Dayasiri

**Affiliations:** 1 Clinical Science, Faculty of Health Care Sciences, Eastern University, Batticaloa, LKA; 2 Clinical Sciences, Faculty of Health Care Sciences, Eastern University, Batticaloa, LKA; 3 Pediatrics, Base Hospital, Mahaoya, LKA

**Keywords:** adolescent pregnancy, batticaloa, sri lanka, low birth weight, prematurity, intrauterine growth retardation, pre- eclampsia, abruptio placenta, apgar, teenage

## Abstract

Background

Adolescent pregnancy is a high-risk condition that can potentially lead to adverse perinatal and obstetric outcomes. It is a growing concern in developing countries including Sri Lanka. The main objective of this study was to evaluate obstetric and perinatal outcomes amongst adolescent pregnancies and compare them with outcomes of pregnancies of average maternal age (AMA) women.

Method

This was a prospective, cross-sectional study conducted at a tertiary care hospital in the Eastern province of Sri Lanka. A total of 795 primigravidae who had singleton pregnancies and delivered their live babies over a period of three months from February to April 2019 were selected for the study. The data were retrieved from Bed Head Tickets (BHTs) of those patients upon discharge from the postnatal ward.

Results

The majority of primigravida was AMA women and accounted for 83.4% (n=663) of the study population while primiparous adolescents comprised 16.6% (n=132). Among the 132 adolescent pregnancies, 81.1% (n=107) were associated with complications and the remaining 18.9% (n=25) were uncomplicated. The most common risk associated with adolescent pregnancy was the low birth weight (LBW; n=24, 18.2%), followed by preterm labour (n=10, 7.6%). An adolescent mother had a 1.3 times higher possibility of delivering an LBW baby than an AMA mother and the risk was statistically significant (p=0.04).

The likelihood of delivering an intrauterine growth restriction (IUGR) baby was higher in adolescent mothers (6%) than in AMA mothers (5%). Adolescent mothers had a 1.2 times higher chance of delivering newborns with IUGR compared to AMA mothers; however, the difference of IUGR was not statistically significant (p>0.05). The incidence of foetal distress was less among adolescent mothers compared to AMA mothers. The majority of the babies (n=126, 95.5%) of adolescent pregnancies were healthy. Only 4.5% (n=6) babies needed neonatal intensive care unit (NICU) admissions; of those, four babies had very LBW and prematurity and the other two babies depressed at birth and developed respiratory distress.

Conclusion

Adolescent pregnancy carries a significant risk of obstetric complications including LBW and prematurity that should draw public and healthcare providers’ attention. The effectiveness of a comprehensive antenatal and community-based program to prevent adolescent pregnancy and related adverse outcomes should further be evaluated.

## Introduction

World Health Organization (WHO) defines adolescent age between 10 and 19 years. Also referred to as “teenage, patients in this category are considered physically and mentally immature to the point where they cannot handle the demands of pregnancy. Therefore, adolescent pregnancy has become a significant social and public health concern mainly in developing countries [1]. More than 90% of adolescent pregnancies worldwide occur in low and middle-income countries [2]. However, adolescent pregnancies are also of concern in the United States; the US has the highest adolescent pregnancy rate of all developed countries, with an annual rate of approximately 8% of total birth [3]. In Sri Lanka, adolescent pregnancies account for 4.4% of all pregnancies as reported in 2019. The percentage of adolescent pregnancies is highest (9.0%) in Trincomalee district followed by Batticaloa district where it was 8.4% during the same period [4].

There are 12 million adolescent females between 15 and 19 years old, and nearly 777,000 adolescent females below the age of 15 years give childbirth annually in unindustrialized nations [5,6]. Further, there are 10 million adolescents in their 15-19 year age, embark unplanned pregnancies in developing countries per annum. Among them, 3.9 million terminate their pregnancies in unhygienic conditions and are attributing to maternal mortality, morbidity, and long-term health issues [5]. This adverse outcome in adolescent pregnancy drew great attention since it has an impact on the biological and socioeconomic status of the country. The adverse outcomes are influenced by their biological immaturity, unintended pregnancy, substandard antenatal care, maternal malnutrition, and anxiety [7].

Amongst 15-19 years old, pregnancy and delivery-related complications are the important causes of maternal mortality and morbidity globally [8]. Adolescent pregnancies have more risk for developing eclampsia, puerperal endometritis, and systemic infections than women between 20 and 24 years, and their newborns, in addition, are more likely to have low birth weight (LBW) and premature which leads to more neonatal complications [9]. Therefore, adverse pregnancy outcome indicators are more in adolescent mothers than AMA mothers. In addition, the infant mortality rate also more in adolescent mothers because of the increased incidence of prematurity among them. The children born to adolescent mothers show poor indicators of health and social wellbeing than the children born to older mothers [10,11].

Certain works of literature show that a higher incidence of undesirable outcomes of adolescent pregnancies could be related to their socio-economic issues rather than the biological age alone. These issues impact their health-seeking behavior [12]. Further, other issues such as under-utilization of existing health services and inaccessibility to antenatal care may also be other contributing factors for poor outcomes in adolescent pregnancy [13].

Despite adjusting all other risk factors for high-risk pregnancies, a handful of studies show a reliable association between adolescent pregnancies and their adverse obstetric outcomes [14]. However, it is unknown the extent of contribution of biological age on outcomes such as LBW, prematurity, and other perinatal complications. Adequate and high-quality antenatal care can uphold good outcomes among adolescent pregnancies [14]. 

## Materials and methods

A prospective and cross-sectional study was carried out in Teaching Hospital, Batticaloa. A total of 795 primigravidae who had singleton live pregnancies and delivered their babies over a period of three months from February to April 2019 were selected for the study. These pregnant women were divided into two categories: (1) average maternal age (AMA) women of 20-35 years old and (2) adolescents between 12 and 19 years old. Among them, 100 mothers with adolescent pregnancy were compared with 100 AMA mothers (20-35 years of age) to determine the foetal and newborn outcomes.

The details of maternal and foetal information were obtained from the Bed Head Tickets (BHTs) of respective mothers upon discharge from the postnatal ward. The maternal information included preeclampsia, gestational diabetic mellitus (GDM), preterm, prelabour rupture of membranes (PPROM), preterm labour, intrauterine growth restriction (IUGR), placenta previa, abruptio placenta, oligohydramnios and polyhydramnios. Furthermore, the details of mode of delivery, lower section caesarian section (LSCS) and its indication, Apgar score at one, five and seven minutes, birth weight, and foetal complications such as LBW, foetal distress, breech presentation, neonatal intensive care unit (NICU) admission and its outcome were collected.

All the information was extracted from BHTs and entered into a standard questionnaire. Data were analyzed using IBM SPSS data package version 24 (IBM Corp., Armonk, NY) and relevant statistical tests were applied. P<0.05 was considered as statistically significant using the Chi-square test.

The protocol for cervical priming and induction of labour was as follows: ripening of cervix was achieved with Foley’s Cather induction or with vaginal prostaglandin E1 (PGE1) tablets given six hours apart. Induction of labour was usually performed with oxytocin infusion following amniotomy at a basal infusion rate of 4 mIU/min, and it was increased by 1 mIU/ min every 15 minutes until it produced four to five uterine contractions every 10 minutes. The maximum applied rate of infusion was 20 mIU/min. The Apgar score had been used according to international standards at birth. Birth weight was assessed using an electronic weighing scale within 15 minutes of birth.

## Results

AMA primigravida accounted for 83.4% (n=663) and 16.6% (n=132) were adolescent primiparous mothers. The mean age of adolescent pregnancies was 19 years. The majority of AMA primigravidae belonged to the age group of 23-24 years and the mean age of AMA mothers was 21 years. Table 1 shows the distribution of the age of mothers in two groups. Among 132 adolescent pregnancies, 107 (81.1%) were associated with complications and the remaining 25 (18.9%) were uncomplicated.

**Table 1:** Distribution of pregnant mother AMA: average maternal age.

Age of Mothers	Frequency	Percent
AMA (20-35 years)	663	83.4
Adolescent (11-19 years)	132	16.6
Total	795	100.0

Table 2 shows the distribution of complications among adolescent mothers. The most common complication associated with adolescent pregnancy was the LBW (n=24, 18.2%), followed by preterm labour (n=10, 7.6%). About 5.3% (n=7) mothers had hypertensive disorders which included (n=5, 3.8%) gestational hypertension and (n=2, 1. 5%) preeclampsia. None was complicated with eclampsia.

**Table 2:** Complications in adolescent mothers GDM: gestational diabetic mellitus, PPROM: prelabour, preterm rupture of membranes, PROM: preterm rupture of membranes, IUGR: intrauterine growth retardation, LBW: low birth weight.

Complications	No. of Mothers	Percent
Hypertension	5	3.8
Preeclampsia	2	1.5
GDM	2	1.5
PPROM	1	0.8
PROM	3	2.3
Preterm	10	7.6
IUGR	6	4.5
Oligohydramnios	4	3.0
Polyhydramnios	1	0.8
LBW	24	18.2
Apgar in 5 minutes <7	3	2.3

Preeclampsia, oligohydramnios and polyhydramnios were found to be higher among adolescent mothers as compared to AMA mothers. Pregnancy-induced hypertension (PIH) and preterm labour were more or less equal in both groups. PIH and oligohydramnios were found to be higher in AMA mothers as compared to adolescent mothers (Table 3).

**Table 3:** Comparison of maternal complications between adolescent and adult mothers PIH: pregnancy-induced hypertension, GDM: gestational diabetic mellitus, DM: diabetes mellitus, PPROM: prelabour, preterm rupture of membranes, PROM: preterm rupture of membranes.

Maternal Complications	Teenage Mothers	AMA Mothers	P-value
PIH	3	3	0.008
Preeclampsia	1	0	
Eclampsia	0	0	
Preexisting DM	1	0	
GDM	2	12	
Placenta praevia	0	0	
Placental abruption	0	0	
PPROM	0	0	
PROM	1	4	
Preterm labour	1	1	
Oligohydramnios	4	3	
Polyhydramnios	1	0	

LBW was observed in a higher proportion of adolescent mothers as compared to AMA mothers and it was also revealed that adolescent mothers had a 1.3 times higher chance of delivering an LBW baby compared to AMA mothers and it was statistically significant (p=0.04).

The percentage of IUGR baby was higher in adolescent mothers (6%) than in AMA mothers (5%); however, the difference of IUGR was not statistically significant (p>0.05). The adolescent mother had a 1.2 times higher chance of delivering an IUGR baby compared to an AMA mother. Foetal distress was less among adolescent mothers compared to AMA mothers (Table 4).

**Table 4:** Foetal complications IUGR: intrauterine growth retardation, LBW: low birth weight.

Foetal Complications	Adolescent Mothers (n=100)	AMA Mothers (n=100)	P-value
Preterm	4	4	
IUGR	6	5	
LBW	19	14	0.0400
Foetal distress	3	9	0.0180
Breech	2	0	0.0260

Regarding mode of delivery amongst adolescent mothers (n=132) in Table 5, the majority (n=109, 82.6%) had normal vaginal delivery (NVD) whilst the rest (n=23, 17.4%) underwent caesarian section; among them, 13 mothers (9.8%) had emergency caesarian section (Em. LSCS) whilst the rest (n=10, 7.6%) underwent elective caesarian section (El. LSCS). Only one baby (0.8%) was delivered by instrument. The indication for caesarian section included foetal distress (n=5, 3.8%), lack of progress or prolonged labour (n=9, 6.8%), malpresentation (n=4, 3.0%), and miscellaneous causes such as absent diastolic flow, antepartum haemorrhage (n=5, 3.8%).

**Table 5:** Mode of delivery among adolescent mothers NVD: normal vaginal delivery, Em. LSCS: emergency caesarian section, El. LSCS: elective caesarian section.

Mode of Delivery	Numbers	%
NVD	109	82.6
Instrumental delivery	1	0.8
El. LSCS	9	6.8
Em. LSCS	13	9.8
Total	132	100.0

The majority of the newborns (n=126, 95.5%) of the adolescent pregnancies were healthy. Only six (4.5%) babies needed NICU admissions, of those, four were preterm and had LBW and two were depressed at birth and Apgar had been less than 7 at five minutes. Only one case (0.8%) of unexplained intrauterine death (IUD) was reported.

## Discussion

Adolescent pregnancy comprises 16.6% of the total pregnancies in this study. This observation is similar to a study in India [6]. More common complications among adolescent mothers in our study were LBW (18.2%) followed by preterm (7.6%), hypertensive disorder (5.3%), IUGR (4.5%), oligohydramnios (3%), foetal distress (3.8%), malpresentation such as breech and left occiput posterior position (n=43%), prelabour rupture of membranes (2.3%), preterm prelabour rupture of membranes (0.8%), polyhydramnios (0.8%) and IUD (0.8%). Apgar score at five minutes is the best indicator for intrapartum hypoxia and the possible negative outcome of the neonate. This study showed the occurrence of intrapartum hypoxia in 2.3% of neonates. Numerous literature in various parts of the globe exposed that preterm labour to be the most common complication and it was estimated to be 10.56% by Dubashi and Wani [15], 13.2% by Sharma et al. [16], and 48% by Seenesh and Shah [17]. The current study showed that there was an equal occurrence of preterm deliveries in both groups (n=10, 7.6%).

The general incidence of LBW among singleton pregnancies was 16.5%, whereas it had been 19% and 14% for adolescent and AMA mothers, respectively. The adolescent mother of having LBW is 1.3 times more than those of the AMA mother. Though there was a significant difference observed in the occurrence of LBW babies between adolescent and AMA mothers, it was not solely attributed to the age of the mothers since other biological and socio-economic variables also could have an effect on LBW such as the body-mass index of the mother, parity, and ethnicity. It is recommended to consider these factors in future researches.

One of the most observed complications among adolescent mothers in other studies was hypertensive disorders, and it had been reported to be 14.2% by Sharma et al. [18], 10.6% by Sarkar et al. [19], and greater than 13.05% by Corcoran [20]. The current study showed a low incidence (5.3%). Hypertensive disorders were higher in the adolescent group whereas diabetes was higher in the controlled AMA group. Although preeclampsia was higher in adolescent mothers, the difference was insignificant (P<0.05) which was the same as in some other studies [21,22]. Therefore, the authors suggest studies with more sample size to conclude on this finding.

The present study agreed with several other studies on the observation of IUGR in two groups as adolescent and AMA mothers. Saxena et al. [23] stated an occurrence of IUGR to be 5.5% in adolescent mothers which was similar to the current study (4.5%). There were two literatures in Egypt and Iran that reported the contrasting findings to the current study as a significant difference in the two groups (P<0.000) [24,25].

The number of vaginal deliveries was more in the adolescent group probably due to the smaller size of the babies. Comparable outcomes were found in other studies [26,27], except in two studies that showed a higher rate of caesarian sections. Variations in the study setting could explain the difference [28,29].

The incidence of caesarian section in this study was 16.6%. Most were done due to the lack of progression (6.8%). It was followed by foetal distress (3.8%), and malpresentation (3.0%). The incidence of caesarian section among adolescent mothers was reported to be 6% by Bhalerao et al. [30], 34% by Mukhopadhyay et al. [27], and 26% by Dubashi and Wani [15]. These studies stated that foetal distress, cephalopelvic disproportion, and contracted pelvis are the important reasons for caesarian section amongst adolescent mothers.

Foetal outcomes in the present study observed that 24 (18.2%) were LBW babies, 6 (4.5%) required NICU admission, and only one (0.8%) was IUD. Some studies in India describe the incidence of LBW babies ranged 33-39% and the incidence of stillbirth ranged 4-5% [7,15]. In the Pacific Islands, the incidence of LBW was 19%.

The incidence of IUGR and LBW in this study was higher in the adolescent group which is consistent with previous studies. The incidence of preterm delivery was equal and foetal distress was more in the adult group. Similar to other studies, the difference was statistically significant [21,26-30]. The greater number of LBW was possibly due to prematurity as four out of the nine LBW babies were born prematurely.

## Conclusions

Though the adverse foetal outcomes seemed to be very low among teenage mothers in our study, teenage pregnancy carries a significant risk of obstetric complications including LBW and prematurity that should draw public and care providers’ attention. The effectiveness of a comprehensive antenatal and community-based preventive program to prevent teenage pregnancy and related adverse outcomes should further be evaluated.
